# Proteome Analysis in PAM Cells Reveals That African Swine Fever Virus Can Regulate the Level of Intracellular Polyamines to Facilitate Its Own Replication through ARG1

**DOI:** 10.3390/v13071236

**Published:** 2021-06-26

**Authors:** Qiangyun Ai, Xiwei Lin, Hangao Xie, Bin Li, Ming Liao, Huiying Fan

**Affiliations:** 1 College of Veterinary Medicine, South China Agricultural University, Guangzhou 510642, China; aqy0716@163.com (Q.A.); seaweedlxw@163.com (X.L.); xiehangao@163.com (H.X.); 2 Research Center for African Swine Fever Prevention and Control, South China Agricultural University, Guangzhou 510642, China; 3 Key Laboratory of Zoonosis Prevention and Control of Guangdong Province, Guangzhou 510642, China; 4 Key Laboratory of Animal Vaccine Development, Ministry of Agriculture, Guangzhou 510642, China; 5 Institute of Veterinary Medicine, Jiangsu Academy of Agricultural Sciences, Key Laboratory of Veterinary Biological Engineering and Technology, Ministry of Agriculture, Nanjing 210014, China; libinana@126.com

**Keywords:** proteomics, ASFV, ARG1, polyamine

## Abstract

In 2018, African swine fever broke out in China, and the death rate after infection was close to 100%. There is no effective and safe vaccine in the world. In order to better characterize and understand the virus–host-cell interaction, quantitative proteomics was performed on porcine alveolar macrophages (PAM) infected with ASFV through tandem mass spectrometry (TMT) technology, high-performance liquid chromatography (HPLC), and mass spectrometry (MS). The proteome difference between the simulated group and the ASFV-infected group was found at 24 h. A total of 4218 proteins were identified, including 306 up-regulated differentially expressed proteins and 238 down-regulated differentially expressed proteins. Western blot analysis confirmed changes in the expression level of the selected protein. Pathway analysis is used to reveal the regulation of protein and interaction pathways after ASFV infection. Functional network and pathway analysis can provide an insight into the complexity and dynamics of virus–host cell interactions. Further study combined with proteomics data found that ARG1 has a very important effect on ASFV replication. It should be noted that the host metabolic pathway of ARG1-polyamine is important for virus replication, revealing that the virus may facilitate its own replication by regulating the level of small molecules in the host cell.

## 1. Introduction

More than a hundred years ago, African Swine Fever (ASF) was first diagnosed in Kenya and gradually spread to Europe and other places [1]. In 2018, the outbreak of African swine fever in China led to the death of a large number of pigs [2], seriously affecting the development of the pig farming industry. The post-infection mortality rate of the disease is close to 100%, and there is no efficient and safe vaccine in the world. The pathogen of the disease, ASFV, containing an envelope, is the only known arbovirus with the DNA genome. Its genome is a single molecule of linear double-stranded DNA (DsDNA), which is about 170 to 190 kbp in size and encodes 150 to 167 open reading frames (ORFs) [3,4]. It was recently reported, by cryo-electron microscope, that the diameter of ASFV BA71V isolates is about 208 nm [5]. ASFV mainly replicates in macrophages and monocytes [6].

After the virus enters the infected cell, it begins to replicate and produce many host–pathogen interactions. Complex interactions between host and infected cells have been shown to lead to changes in specific host cell processes, such as immune response, apoptosis, and inflammation. After ASFV invades cells, its encoded protein will play some roles, such as inhibiting the immunosuppressive effect of PAM cells. The DP96R inhibits type I IFN expression and NF-κB activation [7]. The I329 protein is a homologous analogue of TLR3, which can inhibit TLR3 from inducing IFN production [8]. ASFV encodes the MGF multi-gene family. Among them, the A276R of the MG360 family can inhibit IRF3 phosphorylation and IFN production [8]. The MGF505-7R (A528R) protein can inhibit the type I and type II IFN signal pathways [8], thus limiting the effects of IFNI and IFNII on the induction of JAK-STAT pathway and the expression of ISGs. ASFV has a strong immunosuppressive effect on the host. Cell death induced by apoptosis is a common response to virus infection. ASFV also encodes some anti-apoptotic proteins. Among them, the A179L may be involved in the inhibition of apoptosis during the whole infection process, but not at the earliest stage, when the virus core enters the cytoplasm [9,10]. The expression of the A224L can activate NF-κB, which may inhibit apoptosis by activating the transcription of some anti-apoptotic genes [11,12]. The EP153R is a C-type lectin protein, which can downregulate the expression of MHC-I, inhibits cell apoptosis, and participates in the process of adsorbing blood cells [13]. It was also reported that no phosphorylation of eIF2 α or increased induction of CHOP was detected in ASFV E70 isolates-infected cells with the deletion of the DP71L gene [14], indicating that ASFV may encode other inhibitors of this pathway. Inflammation is a form of resistance of the body to foreign pathogens. The A238L protein encoded by ASFV can inhibit inflammation, but the deletion of the A238L gene does not reduce virus replication in macrophages, nor does it reduce the virulence for pigs [15]. In addition, it was found that the virus-encoded protein L83L could specifically bind to the host gene IL-1β, although it was a non-essential gene [16]. At present, there is no detailed description of the inflammation caused by ASFV infection, but ASFV infection can cause a strong inflammatory response in pigs, so it is necessary to further study the mechanism of inflammatory response caused by ASFV. The pathological changes caused by ASFV are typically characterized by bleeding in multiple organs. Studies have shown that the hemorrhagic damage caused by ASFV is mainly caused by monocytes or macrophages [17]. ASFV infection also leads to severe pulmonary edema, and alveolar edema observed in the final stages of acute and subacute ASF (which is also the main cause of death) is the result of activation of pulmonary intravascular macrophages [18].

At present, the molecular mechanism of African swine fever has not been thoroughly studied, and there is an urgent need to reveal the molecular determinants of the disease. Viruses may have developed a variety of strategies to regulate cellular processes, such as signal pathways and morphology, to promote host cell infection. Instead, cells activate the immune system during infection. The interaction between the virus and host cell is extremely complex, so proteomics is needed to gain insight into the changes in the protein spectrum of the host cell after virus infection. In a previous study, researchers used two-dimensional electrophoresis to study the changes in peptides after ASFV infection [19], and then, based on this technique, it was found that there were 12 altered proteins after ASFV infection in Vero cells [20]. It has been reported that quantitative mass spectrometry was used to identify the difference in host protein spectrum caused by ASFV OURT88/3 strain after infecting WSL-HP, HEK293, and Vero cells [21]. Recently, peptide mass fingerprinting has been used to identify the difference in the host protein change spectrum caused by pigs infected with virulent EV71 and attenuated EV71V, respectively [22]. Different from the above, because the prevalent ASFV strains in China are mainly Genotype II, it is necessary to use quantitative proteomics technology to identify the protein map after infection with host cells by ASFV strains prevalent in China. Quantitative proteomics, combined with TMT, has been widely used to better characterize and understand virus–host-cell interactions [23]. Bioinformatics analysis of protein networks and pathways predicted that ASFV-infected PAM cells could induce signal cascades.

There are many host proteins and small molecular substances that play an important role in virus replication. We need to screen out host proteins that may be related to virus replication and pathogenicity through the proteome, and conduct further detailed studies to confirm which host factors are responsible for ASFV replication.

Arginase (includes spermine, spermidine, and putrescine) is a very important enzyme in the host [24]. Its substrate is L-Arginine and the product is ornithine. It is the primary raw material for the synthesis of polyamines [25]. Polyamines can be beneficial for microorganism survival. In recent studies, it was found that the depletion of polyamines in the host cell inhibits the replication of Bunyavirus (SFTSV) [26]. Previous studies have also reported that Bunyaviruses infection will be accompanied by a decrease in Arginine, causing severe fever with thrombocytopenia syndrome [27].

We found significant changes in the ARG1 gene from the proteomics data. At the same time, further verification is established. The intracellular polyamine levels increased after ASFV infects PAM cells. After the knockdown of ARG1 and the addition of exogenous L-Arginine, ASFV replication was affected, proving that the ARG1-polyamine pathway is very important for ASFV replication. These results suggest that the ARG1-polyamines pathway contributes to the infectivity of ASFV, uncovering a case where the ASFV hijacks host metabolism to facilitate its own replication.

## 2. Materials and Methods

### 2.1. Cells and Viruses

SPF piglets ranging from 3 to 5 weeks of age were bled, the trachea was ligated, and their lungs were removed aseptically. Firstly, the outer surface of the lungs was washed with 0.9% saline, and 100–150 mL of PBS was infused from the trachea into the lungs. After 1–2 min of lavage, the lavage solution was recovered and the above operation was repeated until the lavage solution was clear. The lavage fluid was collected and centrifuged at 2000 r/min for 10 min, then the porcine alveolar macrophages (PAM) precipitate was collected for its next use. After being washed twice, it was cryopreserved for storage. 

The ASFV strain GZ201801 (GenBank: MT496893.1), which belongs to Genotype II, isolated from a pig farm in the Huangpu District, Guangzhou, Guangdong Province of China, was grown on a PAM cell monolayer. The virus was propagated on primary cultures of PAMs. Prior to infection, 2 × 10^6^ PAM cells per well, in a 6-well cell culture cluster, were cultured for 2 days in RPMI-1640 culture medium (Life Technologies, Shanghai, China) supplemented with 10% FBS and 1× antibiotics-antimycotic (Gibco, Shanghai, China), in a 37 °C 5% CO_2_ incubator. Then, the PAM cells were washed twice with 1640 medium, and inoculated by 100 µL of virus supernatant and 900 µL 1640 medium. After 1.5 h of incubation at 37 °C, 2 mL of 10% FBS 1640 medium was added to the wells, and placed in a cell culture incubator at °C. When 80% CPE was observed, the flask was freeze–thawed three times. The supernatant was collected for the next propagation or stored at −80° for experiments. The virus titer is calculated at 50% tissue culture infection dose per milliliter (HAD_50_/mL). All experiments were performed in triplicate.

### 2.2. Growth Curve Analysis of ASFV 

The PAM cells were seeded into plates and cultured in a cell incubator. After being cultured for 1 day, the cells were washed twice with 1640 medium and infected with ASFV at a multiplicity of infection (MOI) of 1. The supernatant was collected from the culture medium at 6, 12, 18, 24, 30, and 36 h post infection (h p.i.). The virus titer was determined by the HAD_50_ method to determine the ASFV virus titer. Then, the virus one-step growth curve was drawn.

### 2.3. HAD_50_ Test Assay

Firstly, 2 × 10^5^ PAM cells were seeded per well in a 96-well plate, with the addition of 200 μL of a cell culture 1640 medium (with a serum concentration of 10%) to each well, and then cultured for 1 d. The virus solution was diluted in a continuous ten-fold ratio, and then added into the 96-well plate with 8 replicates in each gradient. After one day, 20 μL of 1% red blood cells were added to each well, and the red blood cell adsorption was observed 16 h post-infection under microscope until 7 days post infection. If there was a large number of red blood cells attached to the surface of white blood cells, forming a rosette or mulberry shape, it was judged as positive. The Reed and Muench method were used to calculate the value of HAD_50_.

### 2.4. Indirect Immunofluorescence Assay

PAM cells seeded into the 24-well plate were infected by ASFV. The supernatant was removed after 24 h. The cells were washed twice with PBS, and then 4% paraformaldehyde was added into the wells and incubated for 10 min at room temperature. The cells were washed twice with PBS, and a 5% skimmed milk powder solution was added to the wells, then incubated at 37 °C for 1 h. The cells were washed twice with PBST, and P30 mouse monoclonal antibody (Frd Bio, Wuhan, China) was added to the wells and incubated at 37 °C for 1 h. The cells were washed three times with PBST, and FITC goat anti-mouse secondary antibody was added into the wells and incubated at 37 °C for 1 h; then, the cells were washed three times with PBST, and the cells were observed under a fluorescence microscope.

### 2.5. Cell Sample Preparation 

For the TMT experiment, PAM cells were grown in an RPMI 1640 medium. After 2 days, these cells were infected or not with ASFV at MOI of 1. The two parallel cells were harvested at 24 h p.i. SDT buffer was added to the sample. The lysate was sonicated (this step can be skipped for protein solution) and then boiled for 15 min. After centrifugation at 14,000 g for 40 min, the supernatant was quantified with the BCA Protein Assay Kit (Bio-Rad, Hercules, CA, USA). The sample was stored at −80 °C.

### 2.6. Protein Digestion

A total of 200 μg of proteins for each sample was incorporated into 30 μL SDT buffer. The detergent, DTT, and other low-molecular-weight components were removed using UA buffer by repeated ultrafiltration. Then, 100 μL iodoacetamide was added, and the sample was incubated in darkness for 30 min. The filter was washed with 100 μL UA buffer, and then washed with 100 μL TEAB buffer. The protein suspension was digested with 4 μg trypsin (Promega, Beijing, China) overnight at 37 °C, and the resulting peptides were collected.

### 2.7. TMT Labeling

A 100 ug peptide mixture was prepared for each sample, and TMT reagent was used to label each sample. Used a high-pH, reversed-phase fractionation kit, gradient elution of TMT-labeled digestion samples was fractionated into 10 fractions by increasing acetonitrile stepwise gradient elution for the next step of mass spectrometry.

### 2.8. Mass Spectrometry

Each fraction was injected for nanoLC-MS/MS analysis. The peptide mixture was added to the reversed-phase trapping column and separated with a linear gradient of buffer B, and the flow rate was controlled to 300 nl/min.

LC-MS/MS analysis was performed on the Q Exactive mass spectrometer for 60 min, and the mass spectrometer was operated in positive ion mode. MS data were obtained by dynamically selecting the most abundant precursor ion. The instrument was run with the peptide recognition mode enabled.

### 2.9. Bioinformatic Analysis

The protein sequences of differentially expressed proteins were retrieved in batches from UniProtKB database (Release 2016_10) in FASTA format. NCBI BLAST was used to locally search the retrieved sequences in the SwissProt database (pigs) to find homologous sequences, from which the functional annotations were transferred to the research sequence. The first 10 explosions were retrieved when the E value of each query sequence was less than 1 × 10^−3^, and Blast2GO10 (Version 3.3.5) was used for GO mapping and annotation. The GO annotation results were drawn by the R scripts.

The FASTA protein sequences of the differentially changed proteins were blasted using the Kyoto Encyclopedia of Genes and Genomes (KEGG) database (http://geneontology.org, accessed on 25 June 2021) and the corresponding KEGG pathway was extracted.

Based on Fisher’s exact test, the entire quantitative protein annotation was used as a background dataset, and GO enrichment and KEGG pathway enrichment analysis were performed on the three aspects (biological processes, molecular functions, and cellular components). This underwent multiple tests to calculate the *p*-value. Only *p*-values below the 0.05 threshold are considered significant for function categories and pathways.

### 2.10. Western Blot Analysis of Candidate Proteins 

In order to verify the previous TMT proteomics results, several identified proteins were randomly selected for Western blot analysis based on their representativeness and the availability of corresponding antibodies. In short, equal amounts of virus-infected and mock-infected cell lysates (20 μg) were boiled in loading buffer for 5 min to denature, and then separated by 12% SDS-PAGE. After the electrophoresis was completed, the protein was transferred to a 0.22 μm nitrocellulose membrane (Bio-Rad, Guangzhou, China) by wet transfer, and blocked with 5% skimmed milk for 1 h at room temperature. The membrane was washed three times with PBST, for 10 min each time, after which the membrane was incubated with rabbit primary antibodies against several selected protein targets, GAPDH (CST, Shanghai, China), at 4 °C overnight. The membrane was washed three times with PBST, for 10 min each time. Then, the membrane was washed in TBS containing 0.05% Tween-20 (TBST) and incubated with DyLight 488 conjugated goat anti-rabbit IgG (Rockland, NY, USA) or conjugated goat anti-mouse IgG for one hour. The membrane was washed again with PBST, and subsequently scanned and visualized using the Odyssey imaging system (LI-COR Biosciences), and the membrane visualized pictures were saved.

### 2.11. Real-Time Quantitative PCR (qPCR)

Quantitative Real-time PCR (qPCR) was conducted to detect host gene mRNA from PAM cells using total RNA extraction kits (Feijie, Shanghai, China) and HiScript Reverse Transcriptase (Vazyme, Nanjing, China), according to the manufacturer’s protocols by Bio-rad CFX Applied System PCR instrument. 

### 2.12. Nitric Oxide Detection

The detection method of nitric oxide utilized the Gris reaction. The specific steps were as follows. A total of 50 μL of each experimental sample was added to the well. A total of 50 μL of sulfonamide solution was added to all the experimental samples and wells containing the dilution series of the nitrite standard reference curve. After these samples were incubated at room temperature for 10 min in the darkness, 50 μL of NED solution (Dingguo, Beijing, China) was added to all wells. These samples were incubated for 10 min at room temperature in the darkness, and purple began to form immediately. The absorbance was measured within 30 min in a plate reader with 520 nm and 550 nm filters, and the amount of NO in each sample was calculated. 

### 2.13. Determination of Intracellular Polyamines Level

The determination of polyamines uses TLC [28]. For all samples, the cells were washed twice, then the cells were scraped off and centrifuged. The pellet was washed with PBS, and the supernatant removed. A total of 200 μL 2% perchloric acid was added to each cell pellet sample. After these samples were incubated overnight at 4 °C, 200 μL 5 mg/mL dansyl chloride (Sigma-Aldrich, Shanghai, China) acetone and 100 μL saturated sodium bicarbonate were added to each 200 μL supernatant or standard sample (spermine, spermidine, and putrescine) to dansylate the polyamines and neutralize the acid. After incubating these samples overnight in the dark at room temperature, 100 μL of 150 mg/mL proline (Sigma-Aldrich) was added to remove excess dansyl chloride. A total of 500 μL of toluene was used to extract the dansylated polyamine. The hair cell polyamine sample was added to the TLC plate (Dingguo, Beijing, China) at small points, and the solution was chromatographed with a 2:3 cyclohexane–ethyl acetate developing solution for 1.5 h. After the TCL plate was dried, it was visually observed by ultraviolet rays with a wavelength of 365 nm, and the TCL plate image was photographed and stored.

### 2.14. The Effect of L-Arginine on ASFV Replication

L-Arginine (MCE, Shanghai, China) was added to PAM cells at various concentrations (2, 5, 10, 20, or 40 mM). After 24 h of pretreatment, PAM cells were infected with ASFV. After 72 h of infection, the cell supernatant was collected to detect the titer of ASFV virus through HAD_50_ assay.

### 2.15. siRNA Transfection

Firstly, 6 pmol siRNA was diluted with 50 μL serum-free medium and 2 μL RFect (primary cell small nucleic acid transfection reagent) was diluted with 50 μL serum-free medium. These were incubated at room temperature for 5 min. After incubation for 5 min, the siRNA diluent was mixed with the RFect diluent (total volume was about 100 μL). These mixtures were mixed gently and incubated at room temperature for 20 min. The mixed cultures were added to the complete medium of the cell well. The culture plates were shaken gently. These plates were incubated at 37 °C with 5% CO_2_ for 72 h to test the inhibitory effect, or perform subsequent virus infection experiments.

### 2.16. Biosafety Statement

All experiments with live ASFV were performed within the biosafety level 3 (P3) facilities.

### 2.17. Statistical Analysis 

The data were expressed as mean and standard deviation. Statistical analyses of the data calculated the *p* value; * was considered a significant variable, with *p* value of less than 0.05, ** indicated *p* value of less than 0.01, and *** indicated a highly significant difference, with a *p* value of <0.001.

## 3. Results

### 3.1. Kinetics of ASFV-Induced Cytopathology in PAM Cells 

**Figure 1:**
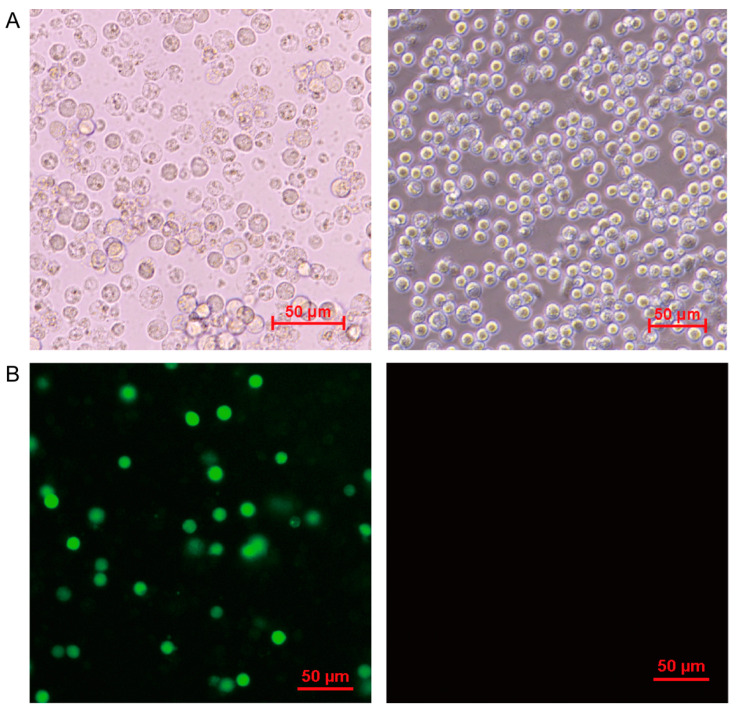
Observation of PAM cells after ASFV infection. (**A**) Typical CPEs caused by ASFV were shown by optical microscope hours 72 h post infection. (**B**) PAM cells were infected by ASFV at MOI of 1. At 24 h post infection, the expression of ASFV encoding protein p30 in PAM cells was detected by indirect immunofluorescence. The left was ASFV-infected PAM cells visual field, the right was mock-infected PAM cells visual field. The red bars in graphs were on behalf of 50 μm.

**Figure 2:**
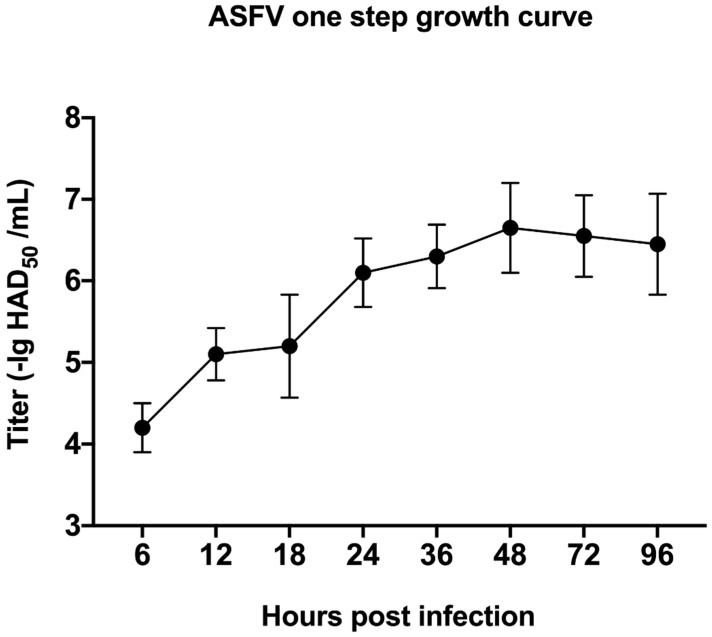
One-step growth curve of ASFV measured through the HAD_50_ end-point dilution assays at 6, 12, 18, 24, 36, 48, 72, and 96 h p.i. Cells were infected with ASFV at MOI of 1. The data are expressed as mean ± SD from triplicate measurements.

We isolated an ASFV strain GZ201801 from the pig farm and successfully and stably reproduced it in PAM cell culture. A significant cytopathic effect (CPE) was observed, which was characterized by the rounding, detachment, and lysis of cells in 1 to 2 days post infection. ASFV infected PAM cells with MOI at 1, and at 6, 12, 18, 24, 30, and 36 h post infection (h p.i.), and the CPE was monitored under a microscope. Other PAM cells were mock-infected. The results showed that, at 24 h p.i., the rounding of the cells and vacuolization can be clearly observed, and the cells were lysed in a large amount at 72 h p.i. (Figure 1A). The mock-infected cells did not show any obvious changes. In addition, IFA indicated that the fluorescence intensity was weak at 12 h p.i. Then, it became stronger within 24 h p.i. (Figure 1B). At the same time, one-step virus growth curve analysis (Figure 2) showed that ASFV production increased steadily in the early stage of infection, and the virus titer reached a peak 48 h after infection.

### 3.2. Cellular Protein Identification and Quantification by TMT Method 

In this study, considering the minimum CPE, 24 h p.i. was selected as the timepoint for TMT proteomics analysis. TMT quantitative proteomics technology was used to carry out the research, and a total of 4218 proteins were identified in porcine Susscrofa domestica. In addition, a large number of viral proteins were identified in the TMT identification results later, including the structural proteins of ASFV p72, p30, p54, etc. (Table S1 sheet 2). The fact that a large number of viral proteins were detected in the host cells 24 h after PAM cells were infected with ASFV indicates that virus reproduction in PAM cells was very active at this time, and it can also be speculated that ASFV has a significant impact on PAM cells at this time. Therefore, it was relatively reasonable to choose 24 h p.i. samples for TMT experiments.

The differentially expressed proteins were screened according to the standard that the expression of multiple changed more than 1.2-fold (up-regulated more than 1.2-fold or down-regulated less than 0.83-fold) and had a *p* value < 0.05. Among them, 306 differentially expressed proteins were up-regulated and 248 were down-regulated (Table S1 sheet 1). Through the analysis of GO function and KEGG pathway, it can be found that the main functions of these differentially expressed proteins are binding, catalytic activity, molecular function regulator, structural molecule activity, and transporter activity, which are mainly involved in important biological processes such as cellular processes, biological regulation, metabolic processes, regulation of biological processes, and response to stimulus.

### 3.3. Verify Protein Changes in TMT Proteomics Data

**Figure 3:**
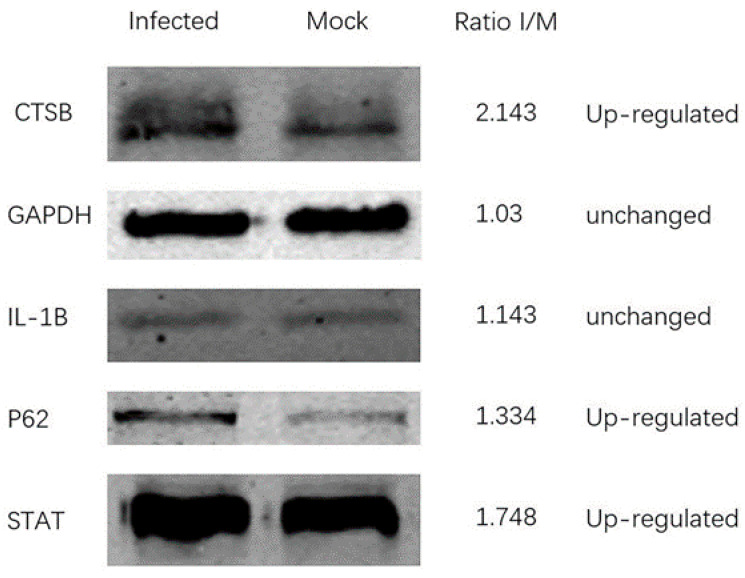
Western blot analysis of host proteins in mock-infected and ASFV-infected PAM cells at 24 h p.i. The TMT ratios (ratio I/M) obtained by mass spectrometry analysis are displayed on the right side.

In order to further confirm the reliability of the TMT quantitative results, according to their representativeness and the availability of corresponding antibodies, five proteins (CTSB, GAPDH, IL-1b, P62, and STAT) were randomly selected for Western blot analysis and verification. They represented up-regulated and unchanged proteins, respectively. As shown in Figure 3, the Western blot analysis of these proteins between ASFV and mock-infected cells was consistent with the trend in the TMT proteomics data.

### 3.4. Functional Classification of Up- and Down-Regulated Proteins in the GO Database 

**Figure 4:**
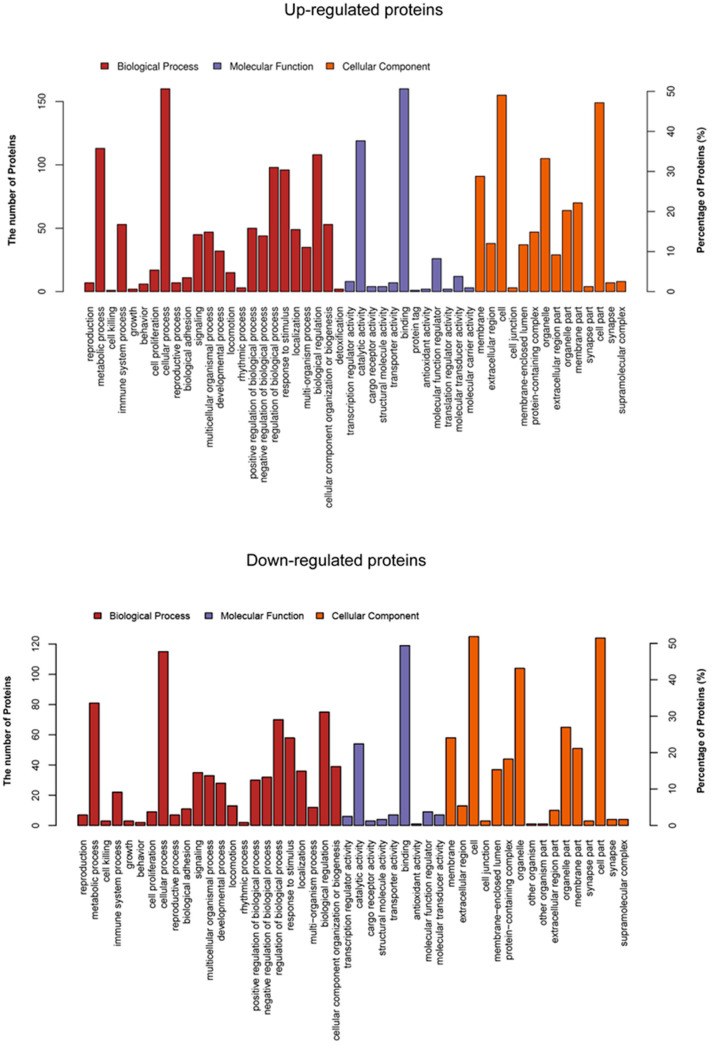
Functional cluster of up- and down-regulated proteins. Biological process, cellular component, and molecular function. Each graph bar indicates the number of the regulated proteins enriched.

The proteins with changes in the proteomics data were analyzed by the software Balst2GO to obtain a functional enrichment analysis based on biological processes and cellular components in the GO database (*p* ≤ 0.05, Table S2). These up-regulated proteins showed that several functional groups are strongly enriched, including immune system processes, defense responses, response to biotic stimulus, and responses to virus by biological process analysis in Figure 4. Meanwhile, the majority of these proteins participated in enzyme inhibitor activity, peptidase regulator activity, and endo-peptidase regulator activity. In the cellular compartment, the up-regulated proteins were localized in the extracellular region and extracellular space. For the down-regulated proteins, the most overrepresented biological processes were involved in the regulation of leukocyte proliferation, regulation of lymphocyte proliferation, and regulation of monocyte proliferation, according to fold enrichment. Meanwhile, the majority of these proteins participated in nucleic acid binding and DNA binding. In the cellular compartment, most proteins were located in the chromosome, nuclear chromosome and MCM complex, etc.

### 3.5. KEGG Pathway Enrichment

**Figure 5:**
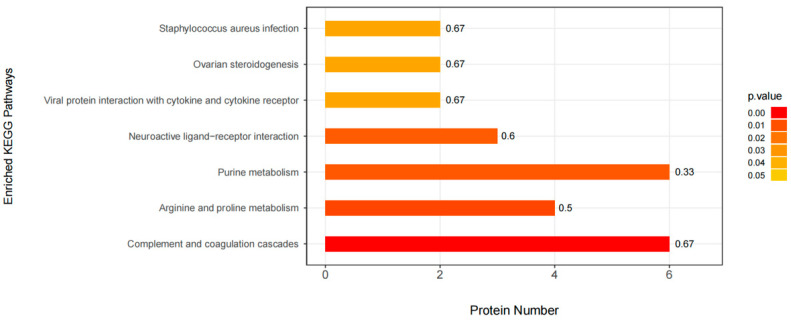
KEGG pathway enrichment analysis. The ordinate represents the significantly enriched KEGG pathway; the Abscissa represents the number of differentially expressed proteins contained in each KEGG pathway; the bar chart color indicates the significance of the enriched KEGG pathway, that is, the *p* value is calculated based on the Fisher accurate test (Fisher’s Exact Test), and the color gradient represents the *p* value. The label at the top of the bar chart shows the enrichment factor (richFator <= 1). The enrichment factor indicates the proportion of the number of differentially expressed proteins involved in a KEGG pathway to the number of proteins involved in the pathway among all identified proteins.

In organisms, there are many different proteins that can coordinate with each other to complete a series of biochemical reactions needed to perform their biological functions. Therefore, pathway analysis is necessary to understand the biological processes, character or disease mechanism, and drug action mechanism of cells more systematically and comprehensively. As shown in Figure 5, the KEGG pathway enrichment of differentially expressed proteins in the comparison group was analyzed by Fisher precise test. The results showed important pathways such as complement and coagulation cascades, arginine and proline metabolism, purine metabolism, neuroactive ligand−receptor interaction, viral protein interaction with cytokine, cytokine receptor, etc.

### 3.6. ASFV Infects PAM Cells to Promote Polyamine Upregulation Through ARG1

**Figure 6:**
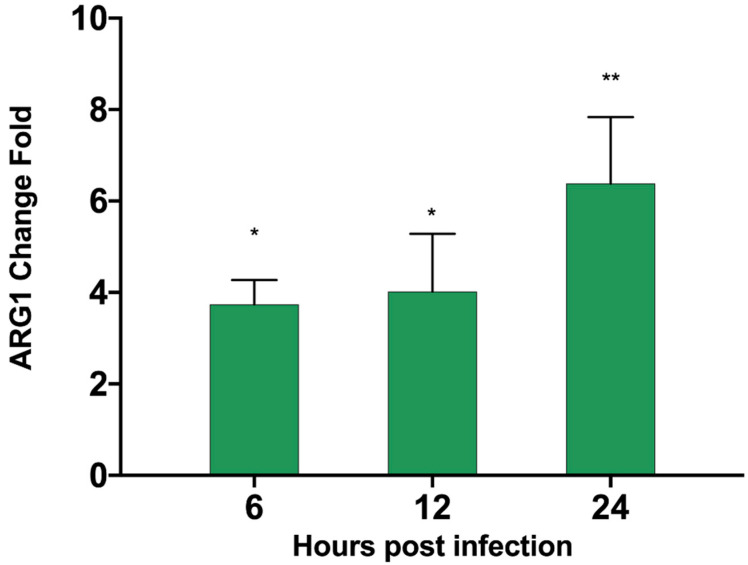
ARG1 mRNA fold change in PAM cells infected by ASFV. PAM cells were infected by ASFV. At 6, 12, and 24 h post-infection, the cells were harvested and mRNA was extracted. The transcription level of ARG1 was detected relative to the mock-infected group. The *p* values were calculated, ** *p* < 0.01, * *p* < 0.05.

**Figure 7:**
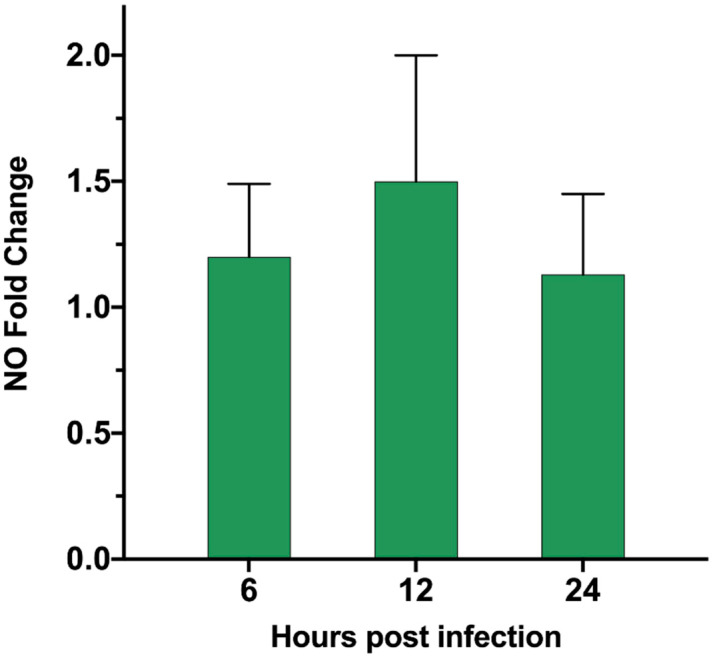
Nitric oxide intracellular level in PAM cells infected by ASFV. PAM cells were infected by ASFV. At 6, 12, and 24 h post-infection, the cell supernatant was harvested, and the change in the amount of NO in the supernatant relative to the mock-infected group was detected.

**Figure 8:**
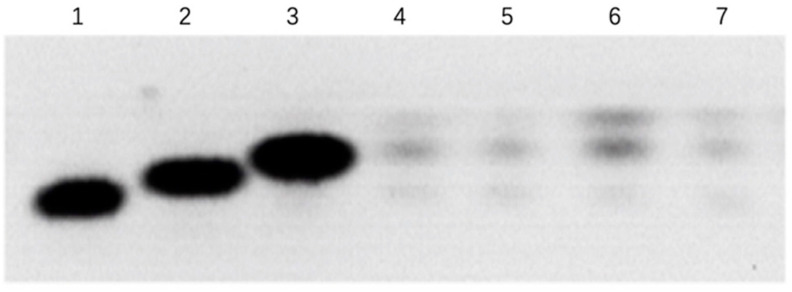
Polyamines intracellular level in PAM cells infected by ASFV. PAM cells were infected by ASFV. At 24 or 48 h post-infection, the cells were harvested and PAM intracellular polyamine levels were detected by the TCL method. Lane (1,2,3) the standard sample of spermidine, spermidine, and putrescine, respectively. Lane (4,6) PAM cells 24 or 48 h p.i. infected by ASFV. Lane (5,7) PAM cells 24 or 48 h mock.

According to our proteomics data, in the up-regulation data, it was found that ARG1 was up-regulated by 1.98-fold, and the *p*-value was less than 0.01 (Table S1 sheet 1). We verified by qPCR that the ARG transcription level (Table 1) was also significantly up-regulated 24 h after ASFV infected PAM cells (Figure 6). The substrate of ARG1 is L-Arginine, which can be used by two enzymes. In addition to ARG1, there is also the iNOs enzyme, which can catalyze L-Arginine to generate nitric oxide (NO). In the proteomics data, this enzyme shows no significant change. We also tested the ASFV infection’s effect on the content of NO in the cell after PAM cells changed, but no significant changes were found. After the detection of ASFV-infected cells (Figure 7), the intracellular small-molecule polyamine levels of downstream product of ARG1 showed significant changes, detected by the TCL method (Figure 8). These results suggest that the ARG1-L-Arginine-polyamine pathway plays an important role in the pathogenesis or replication of ASFV.

### 3.7. L-Arginine Can Promote ASFV Replication

**Figure 9:**
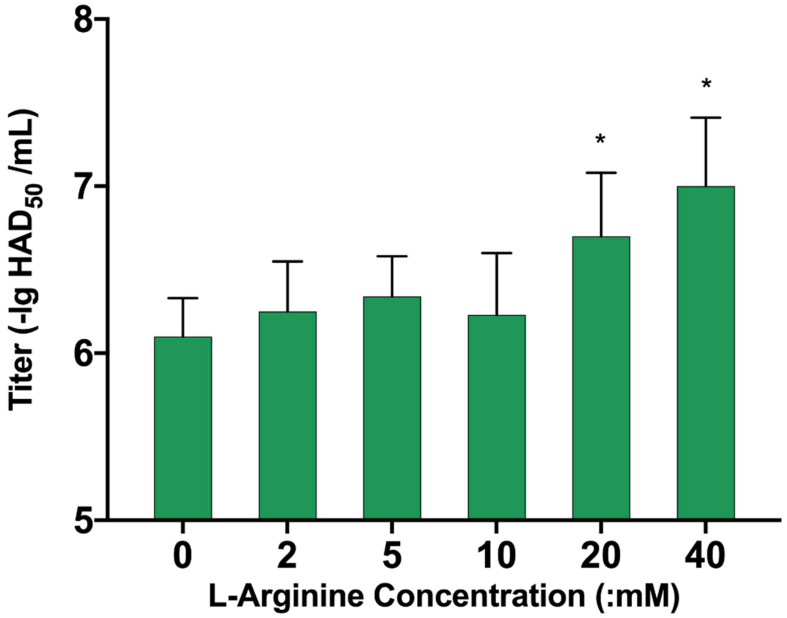
The influence of different concentrations of L-Arginine on ASFV replication. PAM cells were pretreated with 2, 5, 10, 20, and 40 mM L-Arginine for 24 h, then PAM cells were infected with ASFV, cell culture supernatant was harvested 72 h post-infection, and the titer of ASFV virus was detected by the HAD_50_ method. The *p* values were calculated, * *p* < 0.05.

We further verified the effect of ARG1-ployamine pathway on ASFV replication. We selected foreign aid to add L-Arginine to PAM cells for 24 h, then infected the cells with ASFV virus, and harvested the medium after 72 h. The HAD_50_ of each group was tested and was found to be more than 20 mM for ASFV. The duplication of serotonin is clearly promoted, and shows a concentration-dependent effect (Figure 9). These results suggest that L-Arginine was also important for ASFV replication.

### 3.8. Knock Down of ARG1 Gene Affects ASFV Replication

**Figure 10:**
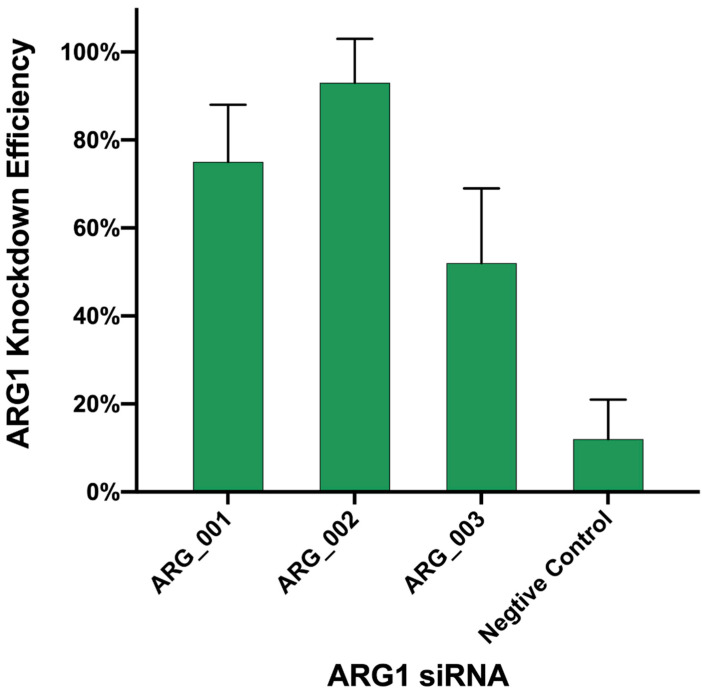
Test for ARG1 siRNA knock-down efficiency. Three siRNAs targeting ARG1 were transfected into PAM cells, respectively, and cell samples were collected 3 days later; the mRNA level of ARG1 was detected, and then the knockout efficiency of ARG1 for each siRNA was calculated.

**Figure 11:**
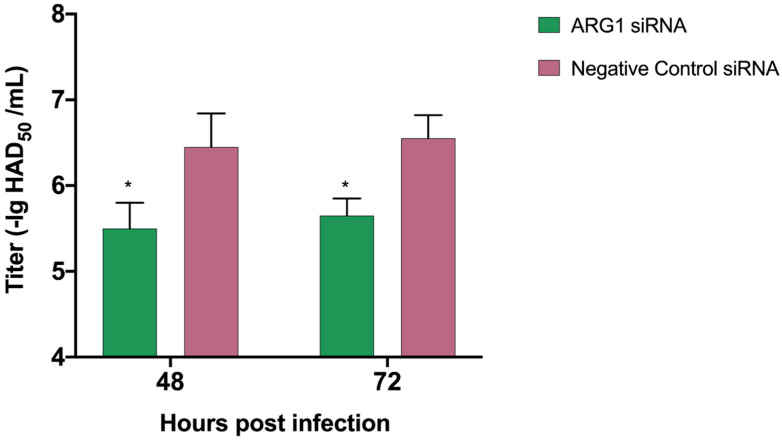
The influence of knockdown of ARG 1 gene on ASFV replication. The siRNA targeting the ARG1 gene with the highest knockout efficiency was transfected into PAM cells. Three days post transfection, PAM cells were infected with ASFV, and the cell culture supernatant was harvested 48 h and 72 h post-infection. The HAD50 method was used to detect the ASFV virus titer. The *p* values were calculated, * *p* < 0.05.

Since no good method was found to transfect PAM cells with overexpression plasmids, we chose to transfect siRNA (Table 2) to knock down the ARG1 gene, to further verify whether inhibiting ARG1 expression affects the replication of ASFV. Firstly, the No. 2 siRNA with the best knockdown efficiency was screened (Figure 10). It was aimed at this sequence. Seventy-two hours after the transfection of PAM cells, ASFV infection was carried out. After 72 h, the virus culture medium was harvested, and it was found that the titer of ASFV decreased (Figure 11). Altogether, our data suggest that the ARG1-L-Arginine-polyamine host metabolic pathway is important for ASFV replication.

## 4. Discussion

As they evolve, viruses may develop a variety of strategies to regulate cell processes to facilitate their own reproduction and spread. However, host cells also can activate the immune system in response to infection due to external stimuli. The interaction between viruses and host cells is quite complex, because it is related to many proteins and cellular processes. The combination of high-throughput quantitative proteomics and TMT technology has been widely used to better characterize and understand virus–host-cell interactions. In this study, we used the TMT method to study the host protein expression changes after ASFV infects PAM cells, and then to study the interaction between the virus and the host, which improved our understanding of the pathogenic mechanism of ASFV and the potential impact of the host on the virus infection. 

Through gene ontology and KEGG pathway enrichment analysis, it has been found that many pathways and biological processes have significantly changed in the histological data, mainly regarding immune system response, complement and coagulation cascade, purine metabolism, arginine metabolism, and the responses of viral proteins and cytokines or cytokines receptor. These host proteins (Table 3) in the related functions pathway mentioned above are discussed in detail below.

We found that the immune response regulated by ASFV is related to immunosuppression, apoptosis, inflammation, etc. Through the changes in several proteins, ASFV regulates the immunosuppression of host cells. As an important connexin, TRAFs initiates signal transduction by interacting with receptors and mediating substrate ubiquitination. TRAF3 is very special in the TRAF family; it neither promotes the activation of classical NF-κB nor activates the MAPK signal pathway [29,30]. TRAF3 is one of the most versatile members of the TRAF family. Through the three signal pathways mediated by TNFR, TLRs, and RLR, TRAF3 plays different functions by participating in different protein complexes [31,32]. It can positively regulate the production of type I interferon and also can negatively regulate MAPK, classical NF-κB, non-classical NF-κB, and mitogen-activated protein kinase (MAPK) signal activation. In addition, knockout of the TRAF3 gene in mice led to early death [33], indicating that the role of the gene is very important for survival, and has a certain immunosuppressive effect. CCL23 showed chemotactic activity in monocytes, resting t lymphocytes, and neutrophils, but not in activated lymphocytes [34,35]. The proliferation of bone marrow progenitor cells was inhibited in the colony formation experiment. In this study, the down-regulation of CCL23 by 0.62-fold indicates the weakening of its chemotactic effect on related immune cells. Sedum heptentokinase (SHPK), formerly known as carbohydrate kinase-like (CARKL), is an enzyme that phosphorylates SedoHeptulose-7- phosphate (sedoHeptulose-7P) [36,37,38,39]. In the screening of new regulators of macrophage activation, it was found that the overexpression of SHPK could block the secretion of tumor necrosis factor-α (TNF-α) induced by lipopolysaccharide (LPS) [39]. SHPK transcripts are down-regulated in mice and humans during LPS-induced inflammation in vitro and in vivo. In vitro, the gene was knocked out by short hairpin RNA (shRNA) modified by microRNAs, resulting in the mild activation of macrophages. In this study, the protein was up-regulated by 1.6-fold, which may inhibit the activation of macrophages and affect immunity to ASFV. PD-L2, encoded by the PDCD1LG2 gene, is the second ligand of PD-1 and can inhibit the activation of T cells [40]. Immune cell infiltration was significantly correlated with PD-L1 and PD-L2 methylation. The inhibition of related immune function by these four proteins, TRAF3, CCL23, SHPK, and PD-L2, may explain the immunosuppressive effect of ASFV on infected host cells.

We also found that ASFV inhibited the apoptosis of infected host cells. In 2004, cytokine-induced apoptosis inhibitor 1 (CIAPIN1) was identified as a newly discovered anti-apoptotic protein [41], which has no homology with Bcl-2 [42], Caspase, and IAP families or signal transduction molecules that regulate apoptosis. CSIG, also known as 1-containing ribosomal L1 domain (RSL1D1), is a kind of nucleolar protein, which contains ribosomal L1 domain at N-terminal and lysine-rich domain at C-terminal [43]. Previous studies have shown that CSIG is involved in a variety of biological processes, including cell senescence and apoptosis [44,45,46,47]. Some studies have shown that CSIG can inhibit the ubiquitination of p53 protein [48], thus inhibiting apoptosis. Both CIAPIN1 and CSIG are up-regulated proteins in the study. BNIP3 is a BCL2-interacting protein 3, which is down-regulated in this study. BNIP3 belongs to the BCL2 family with pro-apoptotic activity [49]. Under hypoxia condition, BNIP3 is up-regulated by hypoxia-inducible factor 1, which initiates LC3-dependent mitosis and prevents overproduction of mtROS [50,51,52]. X-linked apoptosis protein-related factor 1 (XAF1), whose expression is also induced by the interferon (IFN) signal [53], is also a tumor suppressor. XAF1 encodes a protein that acts as an antagonist of the apoptosis inhibitory protein (IAPs), so it also plays a role in promoting apoptosis. In sum, the up-regulation of the apoptosis inhibitor CIAPIN1, CSIG, and the down-regulation of the apoptosis-promoting factor BNIP3 show that ASFV inhibits host cell apoptosis. We note that the apoptosis-promoting factor XAF1 is also up-regulated, but the key molecule of apoptosis-related factors, Caspase-1, is down-regulated by 0.75-fold in this study. Generally speaking, ASFV inhibits host cell apoptosis, which is in line with the replication law of virulent ASFV strains. Inhibiting apoptosis in the early stage of infection can promote the reproduction of the virus itself.

In addition, we also found that ASFV promotes host cell inflammation in non-classical ways thorough some host factors and up-regulates pro-inflammatory factors such as DDX58, in which DDX58 can regulate IL-6 secretion. Apolipoprotein H (APOH) is an acute human plasma protein that has previously been shown to interact with viruses, lipopolysaccharide (LPS), and bacterial proteins [54]. The main functions of APOH are participation in inflammation and protein activation cascade. Apolipoprotein H has been shown to interact directly with a variety of different viruses, such as hepatitis B, rotavirus, and human immunodeficiency virus. 5-Chlorouracil (CLU) is a halogenated DNA base. Like some oxidized DNA bases, it is a nucleic acid reaction product caused by an inflammation-mediated reaction species [55,56,57]. In halogenated uracil, CLU is the most likely product of inflammation-mediated damage. In this study, CLU was up-regulated by 1.54-fold, and some other inflammation-related proteins, such as RSAD2, C5AR1, and TNFAIP6, CAMK2D, were up-regulated by 3.2-fold, 1.34-fold, and so on, indicating that inflammation was indeed activated. The up-regulation of these pro-inflammatory factors showed a pro-inflammatory effect, indicating that the severe inflammatory response of pigs with ASFV is related to this. However, we noticed that there was no significant change in IL-1b, at least in 24 h p.i., so there may be other pathways that activate related inflammatory signals.

In the enrichment results of KEGG pathway, the change in the coagulation cascade reaction pathway is the most significant. ASFV infection of PAM cells showed a great response of coagulation cascade pathway, and significantly up-regulated coagulation-related factors KNG1, PROC, and F9, of which F9 is a coagulation cascade reaction factor 9, which can directly participate in the promotion of blood coagulation [58], while KNG1 can promote inflammation and stimulate vascular dilatation [59,60], and PROC can also cause vascular dilatation and increase endothelial cell permeability [61]. This may be related to cortical ecchymosis, splenic hyperemia, extensive bleeding in various organs, and the corresponding inflammation caused by ASFV, which leads to the coagulation cascade reaction and death of pigs.

The enhancement of purine metabolism will increase the synthesis of uric acid (the oxidative metabolite of purine), resulting in hyperuricemia. When the concentration of serum uric acid is too high, uric acid is deposited in joints, soft tissue, cartilage, and kidney in the form of sodium salt. The tissue foreign body inflammatory reaction can cause gout. ASFV infection promotes the purine metabolic pathway, so it may cause arthritis and glomerulonephritis.

Interestingly, we found a change in alveolar-associated proteins. Swine alveolar surface protein B is a kind of pulmonary surfactant protein, which is 0.44-fold lower than that of the control group in this study. SFTPB protein is hydrophobic, highly expressed in the nucleus and endoplasmic reticulum, and widely distributed extracellularly. Due to endocytosis, the extracellular SFTPB protein is swallowed into the cytoplasm to form endosomes, which fuses with lysosomes after the formation of secondary endosomes, resulting in the appearance of SFTPB proteins in lysosomes [62]. In cells, SFTPB monomers exist in the form of polycysts and are packaged to form lamellar bodies [63]. They enter the secretory pathway by way of vesicle exocytosis, and then play an efficient role in anionic phospholipids such as phosphatidylglycerol, promote lipid fusion and the formation of multilayer lipid structure, and form a phospholipid monolayer at the alveolar gas–liquid interface, effectively reducing the alveolar surface tension and playing an important role in maintaining the normal physiological function of alveoli in lung injury [64]. Knockout of the SFTPB gene can lead to fatal respiratory failure and atelectasis in mice. SFTPB not only reduces the alveolar surface tension, but also has certain antibacterial activity and protects the lung from hypoxic lung injury. Some experiments have shown that knockout of SP-B gene can lead to fatal respiratory failure and atelectasis in mice [65]. SFTPB can not only reduce the alveolar surface tension, but also has certain antibacterial activity and protects the lung from hypoxic lung injury. The dyspnea caused by ASFV and lung failure [66] may be related to this cause. Further research is also needed to determine how SFTPB is affected or regulated by ASFV.

When antiviral immune response occurs, the body strives to strike a balance between activating antiviral immune response and limiting excessive immune response in order to minimize immune-mediated pathology and repair tissue damage. Arginine metabolism plays an important role in immunity and virus infection [67]. On the one hand, arginine is the substrate of arginase and nitric oxide synthase, and its metabolite is an important regulator of T cell function; on the other hand, nitric oxide produced by arginine through nitric oxide synthase regulates platelet function and activation. Hypoarginemia is a potential mechanism leading to blood coagulation disorders. Abnormal arginine metabolism is also a potential mechanism leading to T cell dysfunction and continuous virus replication [27]. In this study, Arg1 is up-regulated, so it will damage the body’s immune response and facilitate ASFV replication.

Through proteomics data, we found that ASFV can cause a significant up-regulation of ARG1 after infecting PAM cells, and this was further verified by qPCR. ARG1 can catalyze the substrate L-Arginine to produce polyamines, and the iNOS enzyme can use the same substrate. In the proteomics data, the expression of this enzyme did not change significantly after ASFV infection, and the intracellular level of its product NO did not change significantly. However, it was found through the TCL method that ASFV infection caused changes in intracellular polyamines level. Further experiments were performed by adding L-Arginine or causing knockdown of the ARG1 gene. It was found that the replication of ASFV can be promoted or inhibited, proving that the ARG1-polyamine host metabolic pathway is important for the replication of ASFV. Arg-1 up-regulation is a hallmark of M2 polarization in PAM cells [68]. M1 but not M2 polarization decreased ASFV ability to replicate in macrophages [69] and these results are in accordance with our data, with ASFV inducing Arg-1 and Arg-1 promoting ASFV efficient replication in its target cells.

There is still a lot of work to be done; the details of related mechanisms need further study to fully understand the importance of this metabolic pathway for ASFV replication.

In addition, the proteomics method, combined with in vitro infection experiments, has led to progress in our understanding of the interaction between ASFV and host cells. Understanding how host factors promote viral replication can provide insight into the basic mechanisms of viral infectivity and highlight novel treatment strategies for ASF.

## 5. Conclusions

In summary, TMT combined with HPLC-MS/MS quantitative proteomics has been studied, looking at the interaction between ASFV and PAM cells. The comprehensive functional analysis of the host proteome after PEDV infection shows that many regulatory proteins are involved in various host cell pathways and biological processes related to immune response and pathogenesis. At the same time, combined with the results of proteomics analysis, further research found that the ARG1-polyamine host metabolic pathway is very important for the replication of ASFV. This study is of great significance for studies on the influence of host metabolism caused by ASFV infection on virus replication. Further research may help us to further understand the pathogenic mechanism of ASFV, better understand the basic mechanism of ASFV infection, and point out new treatment strategies.

## Figures and Tables

**Table 1:** Primer information for qPCR. Design primers to detect the transcription of porcine ARG1 gene in PAM cells.

Primer	Sequence
ARG1-qF	TGTCTTCCGTTCAGTAGGTGG
ARG1-qR	TACACCAGAGTCCTCCAGCC

**Table 2:** The siRNA target sequence for the porcine ARG1 gene. Three siRNA targets were designed with the swine ARG1 gene (Accession number: XM_021082739).

Target Gene	Target Sequence
ARG1_001	GTGGCAGAAATCAAGAAGA
ARG1_002	CCTGAAACCACCTAAGTAA
ARG1_003	GCTATCTACTAGGAAGAAA

**Table 3:** Part of host proteins that are up- or down-regulated in ASFV infected cells. These host proteins may be related to virus replication and pathogenicity.

Gene	I/M Ratio ^1^	*t*-Test *p*-Value
	**Up regulated**	
RSAD2	3.220821	3.7108 × 10^−5^
DDX58	2.410459	0.00020296
ARG1	1.988772	0.00427627
F9	1.797021	1.8209 × 10^−6^
XAF1	1.752608	0.00781031
KNG1	1.665106	0.00626593
SHPK	1.608692	0.01255248
APOH	1.594255	0.01519234
CIAPIN1	1.569497	0.00012041
CLU	1.549828	0.00127142
TRAF3	1.543032	0.00051584
PD-L2	1.402388	0.00099183
C5AR1	1.343329	0.00063514
PROC	1.320784	0.00276507
CAMK2D	1.288763	0.00040577
TNFAIP6	1.210334	0.0297009
**Down regulated**		
SFTPB	0.60814	0.01322343
CCL23	0.623738	0.0098507
BNIP3	0.74174	0.00138866
RSL1D1	0.819272	0.01253252

^1^ I/M ratio (Infection/mock raion). I/M ratio > 1, means up regulated. I/M ratio < 1, means down regulated.

## Data Availability

Data available in a publicly accessible repository.

## Supplementary Materials

Supplementary materials can be found at https://www.mdpi.com/article/10.3390/v13071236/s1. Table S1: Comprehensive proteome map of ASFV-infected PAM cells. Table S2: GO characterization of up- and down-regulated proteins by software.
