# Supplementary figures and images for: Proteome Analysis in PAM Cells Reveals That African Swine Fever Virus Can Regulate the Level of Intracellular Polyamines to Facilitate Its Own Replication through ARG1
Source: Viruses. 2021 Jun 26;13(7):1236. doi: 10.3390/v13071236 (PMC8310191; doi:10.3390/v13071236)

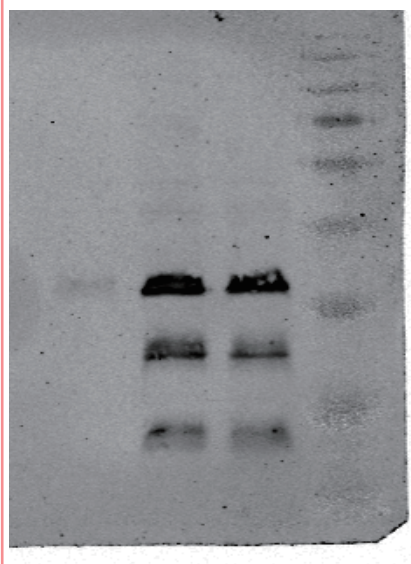

CTSB

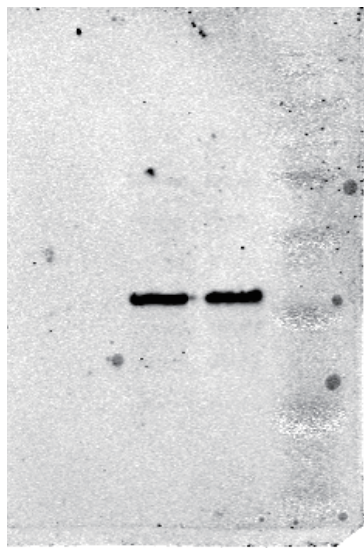

GAPDH

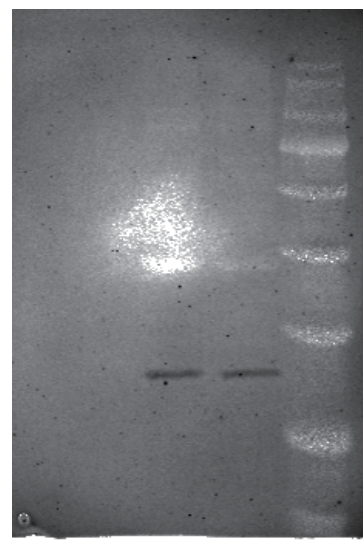

IL-1B

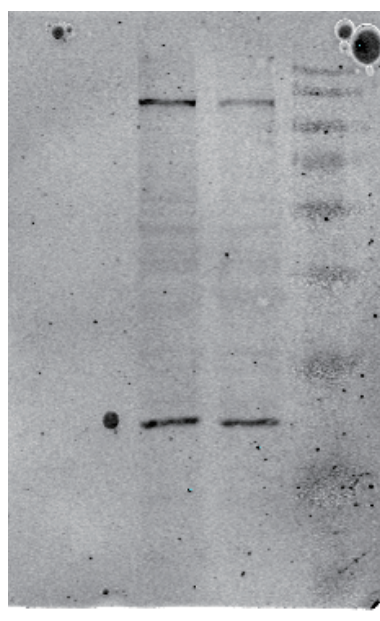

P62

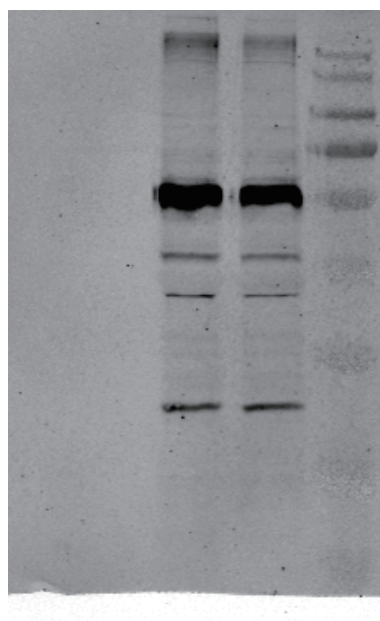

STAT

Figure 3. Western Blot images

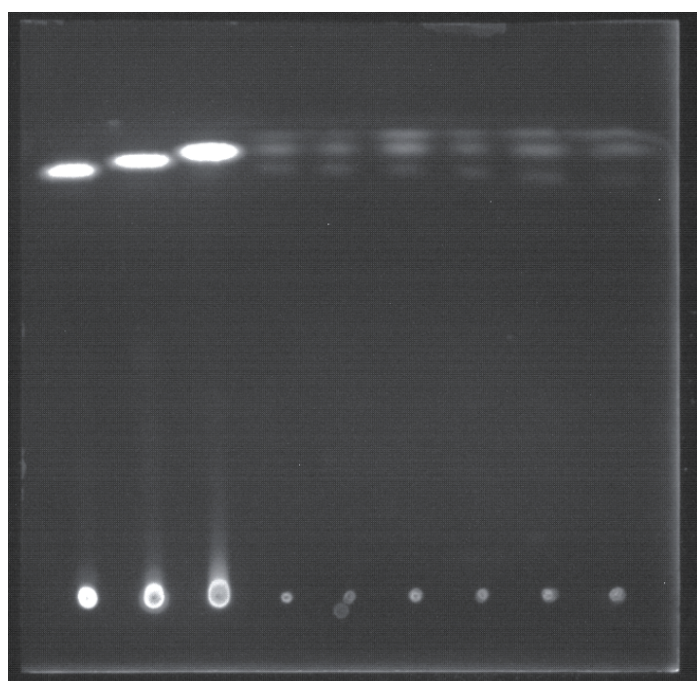

Figure 8. TLC image

Supplement: Supplementary file 1 [file viruses-13-01236-s001.zip › supplementray/Western Blot images TCL original20240624.pdf]
